# Statistical Modeling and Optimization of the Drawing Process of Bioderived Polylactide/Poly(dodecylene furanoate) Wet-Spun Fibers

**DOI:** 10.3390/polym14030396

**Published:** 2022-01-20

**Authors:** Daniele Rigotti, Giulia Fredi, Davide Perin, Dimitrios N. Bikiaris, Alessandro Pegoretti, Andrea Dorigato

**Affiliations:** 1 Department of Industrial Engineering and INSTM Research Unit, University of Trento, Via Sommarive 9, 38123 Trento, Italy; davide.perin-1@unitn.it (D.P.); alessandro.pegoretti@unitn.it (A.P.); andrea.dorigato@unitn.it (A.D.); 2 Laboratory of Polymer Chemistry and Technology, Chemistry Department, Aristotle University of Thessaloniki, 54124 Thessaloniki, Greece; dbic@chem.auth.gr

**Keywords:** fibers, poly(lactic acid), furanoate polyesters, drawing, response surface methodology, genetic algorithms

## Abstract

Drawing is a well-established method to improve the mechanical properties of wet-spun fibers, as it orients the polymer chains, increases the chain density, and homogenizes the microstructure. This work aims to investigate how drawing variables, such as the draw ratio, drawing speed, and temperature affect the elastic modulus (E) and the strain at break (ε_B_) of biobased wet-spun fibers constituted by neat polylactic acid (PLA) and a PLA/poly(dodecamethylene 2,5-furandicarboxylate) (PDoF) (80/20 wt/wt) blend. Drawing experiments were conducted with a design of experiment (DOE) approach following a 2^4^ full factorial design. The results of the quasi-static tensile tests on the drawn fibers, analyzed by the analysis of variance (ANOVA) and modeled through the response surface methodology (RSM), highlight that the presence of PDoF significantly lowers E, which instead is maximized if the temperature and draw ratio are both low. On the other hand, ε_B_ is enhanced when the drawing is performed at a high temperature. Finally, a genetic algorithm was implemented to find the optimal combination of drawing parameters that maximize both E and ε_B_. The resulting Pareto curve highlights that the temperature influences the mechanical results only for neat PLA fibers, as the stiffness increases by drawing at lower temperatures, while optimal Pareto points for PLA/PDoF fibers are mainly determined by the draw ratio and the draw rate.

## 1. Introduction

Biopolymers, i.e., polymers that are bioderived and/or biodegradable, are a promising alternative to traditional plastics, as they reduce the environmental impact of plastic products across the whole life cycle, from resource extraction to end-of-life management [1]. The ever-growing interest in this topic has recently encouraged the development and commercialization of many bioderived, recyclable, and compostable plastics, such as poly(lactic acid) (PLA) [2], polyhydroxyalkanoates (PHAs) [3], thermoplastic starch [4], and poly(butylene succinate) (PBS) [5], which were all employed especially for packaging and other single-use applications.

Among all bioplastics, one of the most diffused is PLA, a biodegradable linear aliphatic poly(α-ester) of lactic acid obtained via biomass fermentation from corn, potato starch, and sugar beets [6,7,8]. The most common grade of PLA on the market is the isomeric form poly-L-lactic acid (PLLA) containing 2–6 wt% of D-lactic acid units [9]. This PLA grade shows elevated tensile modulus (3–4 GPa) and strength (40–60 MPa), high workability, and good transparency [8,9,10]; therefore, it is widely commercialized in the packaging field. PLA is also promising to produce sustainable textiles. Its aptitude to be processed into continuous filaments and nonwovens is being exploited to produce biodegradable textiles for disposable medical and hygienic tissues, but also for technical applications in the filtration and agriculture fields [6,11]. However, the poor toughness, high hygroscopic nature, and high gas permeability of PLA restrict its industrial applications to rigid thermoformed packaging, whereas a wide application of PLA as flexible packaging and textile fibers is still limited [7,12].

To address PLA’s drawbacks and expand its applications, a diffused and cost-effective approach is physical blending, which does not require chemical modifications or the synthesis of new macromolecules [13]. PLA has been blended with several biobased and petrol-based polymers to enhance the strain at break and gas barrier performance [14]. An attractive group of biopolymers that could be blended with PLA is that of poly(alkylene furanoate)s (PAFs). PAFs are produced from the polycondensation of a monomeric unit formed by the reaction between a diol with 2,5-furandicarboxylic acid (FDCA), which was included by the US Department of Energy among the top twelve molecules with high added value derived from sugar biorefinery in 2004 and 2010 [15,16]. PAFs, representing a sustainable and bio-based alternative to terephthalate polyesters, feature superior thermomechanical and gas-barrier properties [17,18,19,20,21], which can be finely tuned by varying the length of the diol chain. Longer alkyl chains in PAFs enhance their molecular mobility, thereby decreasing their glass transition and melting temperatures ($T_{g}$ and $T_{m}$) and improving their ductility [20,22].

Despite the potentialities and the versatility of PAFs, very few publications dealing with PLA/PAF blends can be found in the open scientific literature. Our group has recently developed blends of PLA and PAFs with different alkyl chain lengths (i.e., with a number of carbon atoms in the diol from 2 to 12), prepared in the form of films and fibers through solvent casting, spinning, and electrospinning [23,24,25,26,27]. Although these blends are generally immiscible, the results showed that the addition of 5–10 wt% of any of the considered PAFs to PLA remarkably increases the strain at break and the fracture toughness, especially for cast films.

For PLA/PAF fibers, our group blended PLA with mid- or long-alkyl-chain PAFs, such as poly(pentamethylene 2,5-furandicarboxylate) (PPeF), poly(octamethylene 2,5-furandicarboxylate) (POF), or poly(dodecamethylene 2,5-furandicarboxylate) (PDoF). Fibers were produced through solution spinning, as this technique allows working with small batches and is, thus, suitable for lab-scale experiments. Moreover, this technique can be implemented at room temperature with the application of low shear stresses, i.e., ideal conditions to avoid transesterification reactions that could take place in PAFs when processed in the molten state [21]. Finally, solution spinning can result in fibers with a wide range of diameters and shapes of the cross-section and is suitable for the incorporation of heat-sensitive additives and drugs, a desirable asset for polymer fibers with potential applications in the biomedical sector [28]. Our work on PLA/PAF fibers showed that any of the considered PAFs is effective in decreasing the water absorption tendency of PLA. Moreover, PLA/PDoF fibers with a PDoF content of 20 wt% showed a remarkable improvement in the strain at break compared to neat PLA (up to +300%), with a marginal decrease in the elastic modulus and tensile strength, especially in the drawn fibers. Drawing is one of the most widely used methods to improve the mechanical properties of spun fibers, as it orients the polymer chains, increases the chain density, and homogenizes the microstructure. This process is generally performed between the glass transition temperature ($T_{g}$) and the melting temperature ($T_{m}$) of the polymer to enhance the macromolecular mobility [29].

The interesting mechanical properties of drawn PLA/PDoF fibers encouraged our research group to perform a more systematic characterization, to investigate how the drawing parameters influence their mechanical properties. Most of the studies found in the literature on synthetic fiber drawing, as reported in a recent work of our group [28], investigate the effect of single drawing variables (e.g., temperature, draw ratio, drawing speed) on the final properties, by following the so-called one-factor-at-a-time (OFAT) principle. However, this approach fails in seizing the drawing process in its complexity and does not highlight possible interactions between the considered variables. Conversely, statistical methods, such as the design of experiment (DOE), analysis of variance (ANOVA), response surface methodology (RSM), and genetic algorithms (GA), demonstrate which process variables (factors) significantly impact the resulting properties and if there are interactions between them [30]. This allows building empirical equations that correlate the material properties with the most influential factors and their interactions.

Therefore, this work aims to apply the statistical methods of DOE, ANOVA, RSM, and GA to understand how the drawing process variables affect the mechanical properties of fully biobased wet-spun fibers constituted by neat PLA and PLA/PDoF blends. The final goal is to assess which variables and/or combinations of variables significantly affect the elastic modulus and the strain at break of these fibers to find the optimal processing conditions that maximize both these properties.

## 2. Materials and Methods

### 2.1. Materials and Methods

Polymeric granules of PLA grade 4032D, with a density of 1.24 g cm^−3^, MFI at 210 °C and 2.16 kg of 7 g/10 min, and a melting point of 155–170 °C, were provided by Nature Works LLC (Minnetonka, MN, USA). Poly (1,12-dodecylene 2,5-furandicarboxylate) (PDoF) was provided by the Department of Chemistry of the Aristotle University of Thessaloniki (Greece) in form of chips. PDoF was synthesized by a two-steps polycondensation of dimethyl furan-2,5-dicarboxylate (DMFD) and 1,12-dodecamethylene glycol and had a $T_{g}$ of −5 °C and a $T_{m}$ of 111 °C, as reported in the work of Papageorgiou et al. [20]. Chloroform (HPLC grade), ethanol (purity 99.9%), and methanol (purity 99.9%) were purchased from Carlo Erba Reagents Srl (Milano, Italy). All materials were used as received, without further purification.

### 2.2. Sample Preparation

#### 2.2.1. Fiber Spinning

Fibers were prepared through a wet-spinning process. Neat PLA and PLA/PDoF (80/20 wt/wt) mixture were dissolved in chloroform and stirred at 40 °C for 3 h at a concentration of 0.15 wt/v. Air bubbles were removed by mild ultrasonication in an ultrasonic bath for 10 min. A Harvard apparatus Model 11 Single Syringe was used to extrude the polymer solution from an 18-gauge needle at 0.007 mL/min in a methanol/ethanol (20/80 wt/wt) nonsolvent bath. Filaments were collected from the bath with two rollers of 10 mm in diameter at a speed of 60 rpm, and no drawing was applied to the fibers during the spinning and collection phases. The solvent was evaporated for 24 h in air and then the fibers were stored in a desiccator. Further details of the production process are reported in our previous work [24]. Table 1 reports the values of tensile elastic modulus (E) and strain at break (ε_B_) of the produced filaments, as resulted from the characterization performed in our previous work [24].

#### 2.2.2. Fiber Drawing

**Figure 1:**
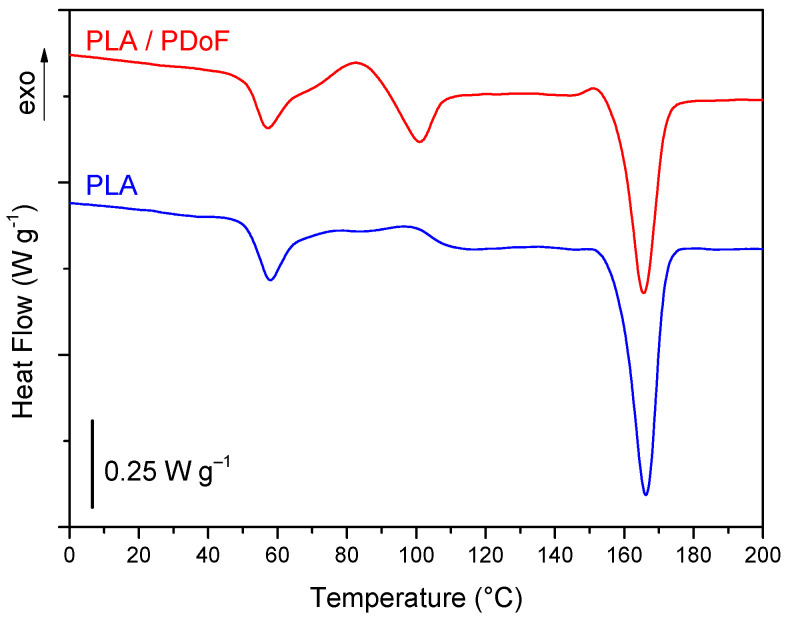
DSC curves of as-spun fibers of PLA and PLA/PDoF (80/20 wt/wt) blend. Data from [24].

To enhance the elastic modulus and strain at break, the as-spun fibers have been drawn. Since the scarce quantity of material available did not allow implementing a continuous in-line drawing, fibers were drawn with an Instron 5969 tensile testing machine equipped with a 100 N load cell and a thermostatic chamber. The drawing was performed at two levels of draw ratio, temperature, and strain rate. The draw ratio was calculated as the ratio between the final filament length and the initial one (50 mm). Draw ratios of 50% and 150% were selected, as these values did not lead to fiber breakage at the selected drawing speeds of 50 mm/min and 100 mm/min. Drawing temperatures were chosen according to DSC data on as-spun filaments of PLA and PLA/PDoF blends reported in previous work (see Figure 1) [24]. A temperature of 40 °C was chosen as it is in between the *T_g_* of PDoF, i.e., −5 °C [20], and that of the PLA used in this work, i.e., 55 °C [24]. Moreover, a temperature of 70 °C was chosen as it lays in between the *T_g_* of PLA and the melting temperature of PDoF, i.e., 103 °C. In this latter condition, both PLA and PDoF are above their glass transition temperature, which should improve the polymer chain mobility.

### 2.3. Characterization

#### 2.3.1. Quasi-Static Tensile Tests

The influence of the drawing parameters of the elastic modulus (E) and the strain at break (ε_B_) of the drawn fibers was evaluated through tensile tests. Quasi-static tensile tests were performed by using an Instron 5969 tensile testing machine equipped with a 100 N load cell. Fiber specimens, with a gauge length of 50 mm, were mounted on paper frame supports and tested at a crosshead speed of 1 mm/min. At least 9 specimens (replicas) were tested for each combination of parameters.

#### 2.3.2. Design of Experiment, Statistical Analysis, and Genetic Optimization

In this study, PDoF was employed to improve the mechanical behavior of PLA-spun fibers. Therefore, careful selection of the drawing parameters is necessary to ensure the achievement of the maximum degree of improvement. Preliminary experiments were conducted to identify the process parameters and their range. Based on these experiments, four process parameters were selected in the study, i.e., draw ratio (x_1_), drawing rate (x_2_), composition of the fiber (x_3_), and drawing temperature (x_4_).

To investigate how these variables influence the values of E and ε_B_ of the fibers, a 2^4^ full factorial design [31] was used, whose factors and levels are given in Table 2. Each factor varies between two levels, normalized between −1 and +1.

The results of the mechanical tests were analyzed with the response surface methodology (RSM) technique to obtain empirical models for E and ε_B_. RSM explores the relationships between several explanatory variables and one or more response variables. The method was introduced by G. Box and K. B. Wilson in 1951. The main idea of RSM is to use a sequence of designed experiments to obtain an optimal response. Although this model is only an approximation, it is very easy to apply even if little is known about the process, and it allows the evaluation of how multiple factors and their interactions affect one or more response variables [32].

Full quadratic response surface was employed to model the influence of the considered factors on the mechanical properties of the fibers, according to the expression reported in Equation (1):

(1) $$y=\beta_{0}+\overset{k}{\underset{i=1}{\sum }}\beta_{i}x_{i}+\overset{k}{\underset{i=1}{\sum }}\beta_{ii}x_{i}x_{i}+\overset{k-1}{\underset{i=1}{\sum }}\overset{k}{\underset{j=i+1}{\sum }}\beta_{ij}x_{i}x_{j}+\epsilon$$

where *y* is the measured output, *x_i_* is the designated input variable, *β_ij_* is the regressor coefficient, and *ε* is the error term.

After this step, an analysis of variance (ANOVA) was used to evaluate the significance of each term on the measured responses. The terms with a high F value (Fisher test) and a low probability value (*p* < 0.05) were selected as the most significant, while the terms with a larger *p*-value (*p* > 0.05) were considered non-significant.

Optimal process parameters, i.e., the combinations maximizing both elastic modulus and strain at break, were identified by the multi-objective non-dominated sorting genetic algorithm II (NSGA2) optimization technique. Genetic algorithm (GA) operates on the principle of natural selection according to the Darwinian theory, in which the strongest species survive and propagate while the least successful disappear. NSGA2 was proposed by Deb et al. [33] and is a popular and powerful multi-objective evolutionary algorithm. NSGA2 has been established as a strong method among the numerous methods of multi-objective optimization in many fields of material science [34,35,36,37].

## 3. Results

Table 3 reports the mean values of elastic modulus and strain at break for the 2^4^ design from tensile tests on drawn filaments.

By comparing these results with those of the as-spun fibers reported in Table 1, it is evident that the drawing process considerably improves the mechanical properties of the fibers, and this improvement is more remarkable in the PLA/PDoF blend than in the neat PLA fibers. For example, the PLA/PDoF blend reaches an elastic modulus of 3.1 GPa (+25% than the as-spun fibers) and a strain at break of 224% (+194% than the as-spun fibers) after drawing at 70 °C with a draw ratio of 50% and a drawing rate of 50%/s. Therefore, the drawing process is very promising to obtain high-performance PLA/PDoF fibers. This is evident despite the high standard deviation of some results, which stems from the non-uniform fiber diameters, the porous microstructure, and the non-complete solvent removal, as explained in our previous work [24]. More specifically, the selected spinning parameters could be more suitable for one composition (e.g., PLA/PDoF) and less for the other (e.g., PLA), and the residual solvent can affect the two polymer phases in a different way, which could partially explain the large standard deviations and the non-significant difference in elastic moduli of the as-spun fibers. However, varying the spinning parameters would have further increased the number of variables, thereby complicating the experimental design.

The response surface analysis on the experimental data of elastic modulus and strain at break resulted in the regression equations presented in Equations (2) and (3), respectively.

(2) $$\begin{array}{l} E=4230.569-199.715\;x_{1}-75.177\;x_{2}-339.260\;x_{3}-208.744\;x_{4}\\ \;\;\;\;\;\;+172.290\;x_{1}\;x_{2}+149.181\;x_{1}\;x_{3}+771.923\;x_{1}\;x_{4}\\ \;\;\;\;\;\;+264.677\;x_{2}\;x_{3}-36.073\;x_{2}\;x_{4}+234.960\;x_{3}\;x_{4} \end{array}$$

(3) $$\begin{array}{l} \varepsilon_{B}=55.2417-16.25\;x_{1}-4.7792\;x_{2}+16.625\;x_{3}+43.0417\;x_{4}\\ \;\;\;\;\;\;+2.1875\;x_{1}\;x_{2}-6.3500\;x_{2}\;x_{3}-21.2\;x_{1}\;x_{4}\\ \;\;\;\;\;\;-7.0542\;x_{2}\;x_{3}-2.7375\;x_{2}\;x_{4}+17.9083\;x_{3}\;x_{4} \end{array}$$

**Figure 2:**
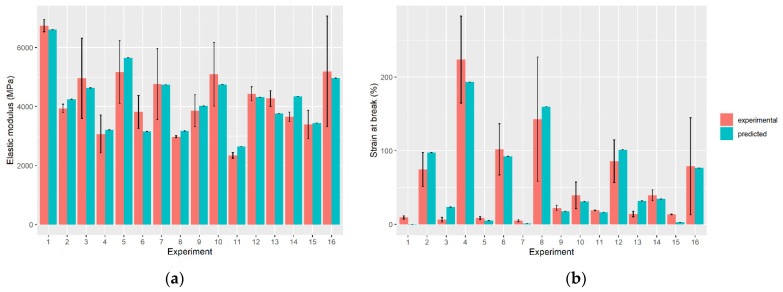
Comparison between experimental and predicted values for (**a**) elastic modulus and (**b**) strain at break of the drawn fibers.

A comparison between the predicted and experimental data of both responses was made to validate the regression models. Figure 2 represents the comparison between data obtained for elastic modulus and strain at break according to Equations (2) and (3), respectively. The obtained results show a good agreement between the experimental and predicted data.

**Figure 3:**
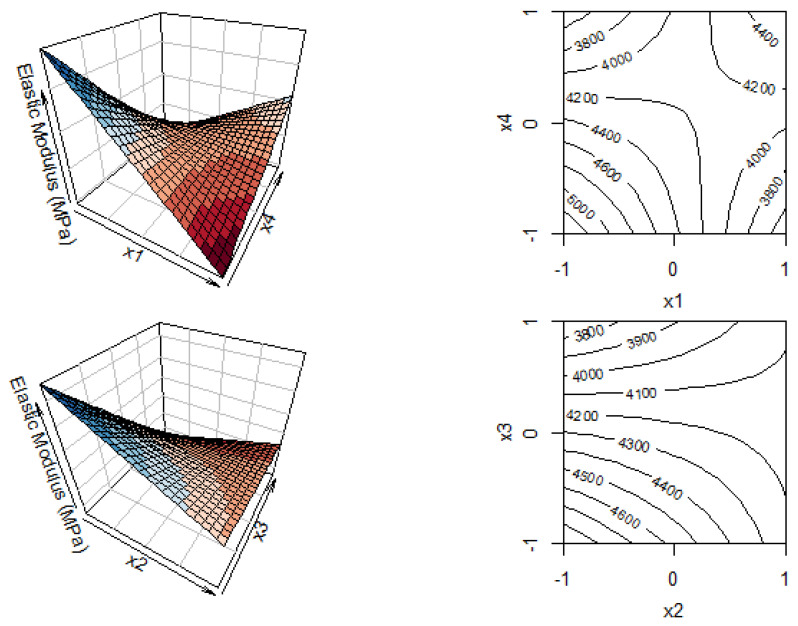
Three-dimensional and two-dimensional plots for the response surface related to the elastic modulus as a function of draw ratio (x_1_) vs. temperature (x_4_) and draw rate (x_2_) vs. PDoF content (x_3_).

ANOVA results of the linear regression of the elastic modulus values (Table 4) evidence a significant effect of only the linear term related to the PDoF concentration (x_3_). According to the *p*-value, interaction effects are observed between the draw ratio and the temperature, as well as between the draw rate and the PDoF concentration. These interactions between variables are shown in Figure 3 as 3D plots and their corresponding contour plots, which also show numerical results. Adjusted R-squared (adj R^2^), also called the coefficient of determination, is a statistical result representing the proportion of the variance for a dependent variable that is explained by an independent variable or variables in a regression model, adjusted for the number of predictors in the model. Adj R^2^ is expressed as a number between 0 and 1, with 1 indicating a perfect correlation and zero indicating no correlation at all. The coefficient of determination is found to be 53.4% in this case.

**Figure 4:**
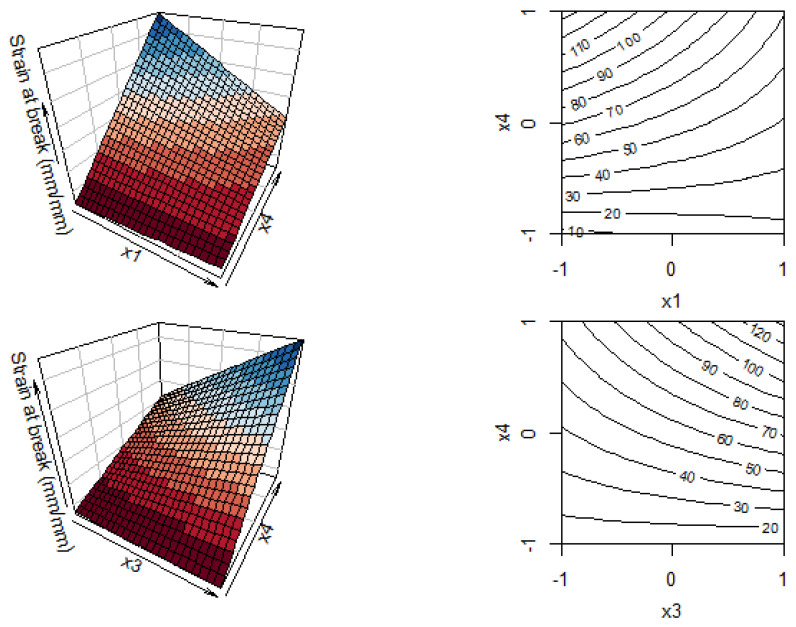
Three-dimensional and two-dimensional plots for the response surface of the strain at break as a function of draw ratio (x_1_) vs. temperature (x_4_) and draw rate (x_2_) vs. temperature (x_4_).

The strain at break, according to the ANOVA results (Table 5 and Figure 4), is found to be significantly affected by the linear terms corresponding to the draw ratio (x_1_), the concentration of PDoF (x_3_), and the process temperature (x_4_). Temperature and its interaction with the draw ratio and with fiber composition are observed to be significant, with a *p*-value < 0.01. In this case, the coefficient of determination is 71.7%.

Stiffness and strain at break are often competitive properties, but if considered together, they represent a first glimpse of the material toughness, and for this application, it would be desirable that both these properties are elevated. To maximize both responses, the regression equations for elastic modulus (Equation (2)) and strain at break (Equation (3)) were combined in the fitness optimization function in R-CRAN software [38], a freely available language and environment for statistical computing and graphics applying the genetic algorithm Nsga2R library.

A genetic algorithm is a search heuristic that is inspired by Charles Darwin’s theory of natural evolution. This algorithm reflects the process of natural selection, where the fittest individuals are selected to produce offspring of the next generation. The progeny inherits the characteristics of the parents, thus further increasing the fitness and the chance of survival. This process iterates until a generation with the fittest individuals is found.

The process begins with a set of individuals called a population. Each individual is characterized by a set of parameters (variables) known as genes, which are joined into a string to form a chromosome (solution). The fitness function determines the ability of an individual to compete with other individuals and gives a fitness score. The higher the fitness score, the higher the probability for an individual of being selected for reproduction. Therefore, the fittest individuals are selected and pass their genes to the next generation.

Based on their fitness scores, two pairs of individuals (parents) are selected. For each pair of parents, a crossover point is chosen at random from within the genes, and this crossover phase is the most significant in a genetic algorithm. In certain new offspring formed, some of their genes can be subjected to a mutation with a low random probability. Mutation occurs to maintain diversity within the population and prevent premature convergence. The algorithm terminates when the population has converged, i.e., it does not produce significantly different offspring from the previous generation. At convergence, the genetic algorithm has provided a set of solutions to the problem [39]. The parameters considered to evaluate the optimal process through genetic algorithm were: population size (100), the size of tournament (2), the number of generations (1000), crossover probability (0.9), crossover distribution index (20), mutation probability (0.1), and mutation distribution index (3) [40]. All the variables were left free to move from the lowest to the highest state (−1, 1).

**Figure 5:**
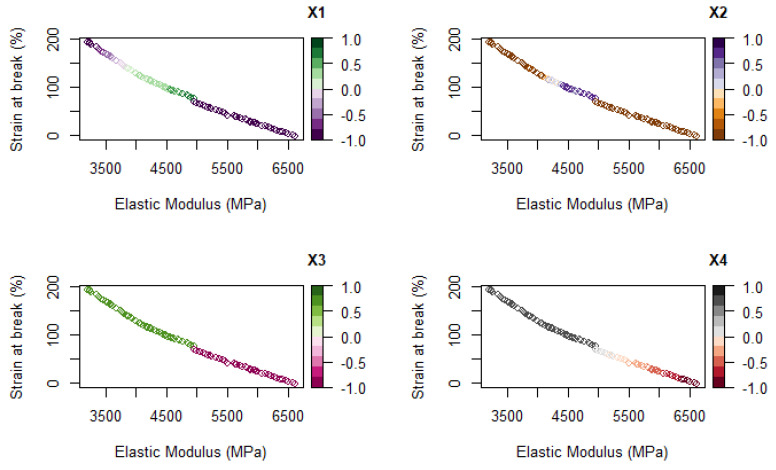
Pareto curves related to the optimal combination between the strain at break and elastic modulus; the changing colors represent the variation in the selected variable.

**Figure 6:**
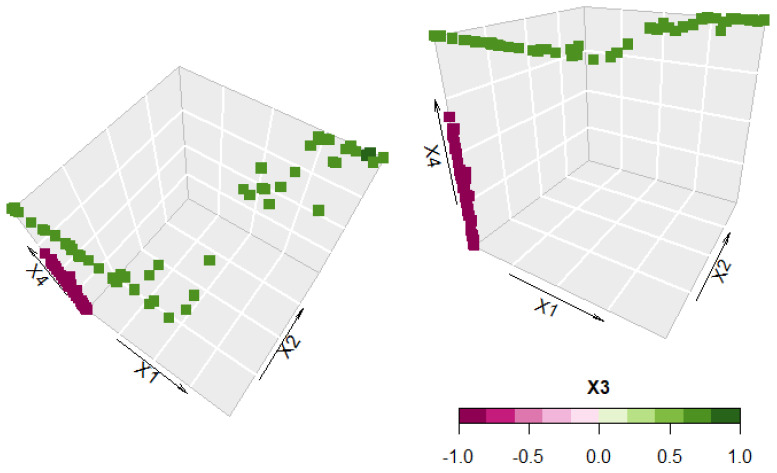
Optimal variables combination from the Pareto curve; color represents the PDoF content as a 4th dimension.

The output of this analysis is a Pareto-optimal front [41], i.e., a set of non-dominated solutions in which no objective can be improved without sacrificing at least one of the other objectives. Figure 5 shows the Pareto curves, where no improvement can be made to one of the two output values, i.e., elastic modulus or strain at break, without reducing the other, with a color palette that represents the value of the considered variable. Each point on the Pareto curve represents an optimal combination of variables, also represented in a 4D space in Figure 6.

## 4. Discussion

By comparing the elastic modulus and strain at break of the fibers before (Table 1) and after (Table 3) drawing, it is evident that drawing is effective to improve the mechanical properties of wet-spun fibers, provided that drawing parameters are carefully selected.

For the response surface of the elastic modulus (Equation (2)), the resulting low R^2^ (0.53) suggests a high scattering of the experimental data. However, the ANOVA analysis reported in Table 4 points out the noticeable effect of PDoF on the mechanical properties. The stiffness of the drawn fibers was impaired by PDoF, which is more likely related to the low stiffness of PDoF rather than the incompatibility between the two polymer phases, given that the strain at break instead increases upon PDoF addition. According to ANOVA, the strongest effect on the elastic modulus was given by the interaction between the draw ratio and the temperature. The lowest draw ratio and the lowest temperature lead to the highest value for the elastic modulus, which could be attributed to the competition between the orientation of the macromolecular chains subjected to stretching and the relaxation of the same chains during stretching, which is hindered at a lower temperature [42]. Similar results were found by Walker et al. for commercial PLA-based extruded filaments [43]. At the highest draw ratio, the decrease in stiffness, but also in the strain at break, could be associated with void generation due to overdrawing, but a deeper microstructural analysis is required to fully explain these findings [44].

Another significant effect is given by the interaction between the drawing speed and the composition. As shown in Figure 3, increasing the drawing speed decreases the elastic modulus of neat PLA fibers but increases it for blended fibers. However, the presence of PDoF decreases the stiffness of the fiber due to its lower elastic modulus compared to neat PLA [22].

For the strain at break, the multi-dimensional response surface is reported in Equation (3) and the graphical representation is shown in Figure 4. In this case, only the curves as a function of x_1_, x_4_ and x_3_, x_4_ are presented, due to their highly significant effects after emerging from ANOVA analysis (Table 5). An increase in the draw ratio reduces the strain at break, due to the enhanced macromolecular orientation, similarly to what has been previously reported by La Mantia et al. [42] for low-density polyethylene/polyamide 6 incompatible blend films and by Gupta et al. [44] for PLA-spun fibers. The drawing temperature has the highest effect (*p*-value = 2.94 × 10^−10^). Drawing the fibers above the glass transition temperature of PLA leads to higher values of strain at break due to the improved strain-induced crystallization and orientation, as well as the microstructural homogenization [45].

Since stiffness and strain at break are often competitive properties, i.e., an increase in one of them is generally accompanied by a decrease in the other, it can be interesting to evaluate which combination of drawing parameters can produce the optimal balance between these two properties. Figure 5 evidences a clear separation between the two compositions, as observable by the curve splitting into two by the variable x_3_ (PDoF content). Fibers of neat PLA can reach higher stiffness at the expense of strain at break compared to PLA/PDoF fibers. Temperature influences the results only for neat PLA fibers, as the stiffness increases by drawing at lower temperatures. Conversely, optimal Pareto points for PLA/PDoF fibers are not influenced by temperature but only by the draw ratio (x_1_) and the draw rate (x_2_). For low values of both these parameters, the highest values of strain at break are obtained. This separation between the behavior of the two components of the blend is well visible in Figure 6, where optimal points for neat PLA are on the x_4_ axis while the ones for the PLA/PDoF blend lie on the x_1_, x_2_ plane at x_4_ = 1.

## 5. Conclusions

The mechanical behavior of PLA/PDoF fibers obtained by wet solution spinning was dramatically improved by the drawing process. Draw ratio, draw rate, composition, and temperature were the parameters taken into account to investigate the mechanical response of the fibers resulting from the drawing process.

The elastic modulus was considerably affected by the composition, as the presence of PDoF, less stiff than PLA, generally decreased the stiffness of the drawn fibers at every level of the other considered factors. Moreover, the elastic modulus was maximized if the temperature and draw ratio were both low, although fairly good values of elastic modulus were obtained when temperature and draw ratio were both high. On the other hand, keeping the temperature high and the DR low, or vice versa, were not ideal combinations of parameters. Therefore, if the drawing temperature is high, the DR must be high, so that chain orientation overcomes the competing mechanism of chain relaxation. The other factors and combination of factors were proven to have little or no significance.

The strain at break was enhanced when the drawing was performed at a high temperature, which was the most influencing factor. This happened likely because high temperature favored microstructural homogenization, pore closure, and the orientation of PDoF domains, which produced a morphology with fewer defects in the drawing direction. Moreover, the strain at break was further enhanced in the PLA/PDoF blends and when the high temperature was coupled with low DR, probably because it prevented excessive microstructural orientation and crystallization.

These results highlighted the impact of the draw ratio, draw rate, composition, and drawing temperature on the final mechanical properties of wet-spun PLA and PLA/PDoF fibers, evidenced by the power of statistical methods, such as ANOVA and RSM, in finding which parameters and combination of parameters are significant, and established guidelines to optimize the drawing process for PLA/PDoF fibers and maximize their stiffness and ductility.

## Figures and Tables

**Table 1:** Elastic modulus and strain at break of the as-spun fibers of neat PLA and PLA/PDoF (20 wt%) blend.

	Elastic Modulus [MPa]	Strain at Break [%]
PLA	2241 ± 377	127 ± 48
PLA/PDoF20	2545 ± 569	76 ± 26

**Table 2:** Selected factors and relative levels for the 2^4^ full factorial design.

Level		−1	+1
Draw ratio (%)	x_1_	50	150
Draw rate (mm/min)	x_2_	50	100
Composition (PDoF wt%)	x_3_	0	20
Drawing temperature (°C)	x_4_	40	70

**Table 3:** Elastic modulus and strain at break values from tensile tests on drawn fibers.

Exp.	Draw Ratio	Draw Rate	PDoF	Temperature	Elastic Modulus	Strain at Break
NO	[%]	[%/s]	[wt%]	[°C]	[MPa]	[%]
1	50	50	0	40	6748 ± 208	9.4 ± 2.2
2	50	50	0	70	3936 ± 142	74.4 ± 23.2
3	50	50	20	40	4959 ± 1359	6.2 ± 3.2
4	50	50	20	70	3068 ± 643	223.8 ± 59.1
5	50	100	0	40	5172 ± 1067	8.5 ± 2.2
6	50	100	0	70	3819 ± 557	101.8 ± 34.7
7	50	100	20	40	4765 ± 1207	4.9 ± 1.5
8	50	100	20	70	2976 ± 37	142.9 ± 84.4
9	150	50	0	40	3861 ± 547	22.3 ± 3.4
10	150	50	0	70	5094 ± 1079	39.3 ± 18.2
11	150	50	20	40	2346 ± 96	19.1 ± 0.5
12	150	50	20	70	4434 ± 236	85.7 ± 28.9
13	150	100	0	40	4274 ± 262	13.8 ± 3.7
14	150	100	0	70	3656 ± 157	39.5 ± 7.3
15	150	100	20	40	3390 ± 481	13.5 ± 0.5
16	150	100	20	70	5192 ± 1881	78.8 ± 65.9

**Table 4:** ANOVA table for the response surface fit of elastic modulus. Df degree of freedom, Sum Sq sum square, F-value, *p*-value, and its significative code: 0 < “***” < 0.001 < “**” < 0.01 “*” < 0.05 “.” < 0.1.

	Df	Sum Sq	Mean Sq	F Value	Pr (>F)	
x_1_	1	1,914,524	1,914,524	2.5995	0.115397	
x_2_	1	271,277	271,277	0.3683	0.547622	
x_3_	1	5,524,686	5,524,686	7.5013	0.009428	**
x_4_	1	2,091,550	2,091,550	2.8398	0.100371	
x_1_:x_2_	1	1,424,818	1,424,818	1.9346	0.172567	
x_1_:x_3_	1	1,068,242	1,068,242	1.4504	0.236107	
x_1_:x_4_	1	28,601,519	28,601,519	38.8343	3.05 × 10^−7^	***
x_2_:x_3_	1	3,362,590	3,362,590	4.5656	0.039298	*
x_2_:x_4_	1	62,460	62,460	0.0848	0.772513	
x_3_:x_4_	1	2,649,907	2,649,907	3.598	0.065677	.

**Table 5:** ANOVA table for the response surface fit of the strain at break. Df degree of freedom, Sum Sq sum square, F-value, *p*-value, and its significative code: 0 < “***” < 0.001 < “**” < 0.01.

	Df	Sum Sq	Mean Sq	F Value	Pr (>F)	
x_1_	1	12,675	12,675	10.36	0.00268	**
x_2_	1	1096	1096	0.8961	0.349972	
x_3_	1	13,267	13,267	10.8436	0.002188	**
x_4_	1	88,924	88,924	72.6824	2.94 × 10^−10^	***
x_1_:x_2_	1	230	230	0.1877	0.667323	
x_1_:x_3_	1	1935	1935	1.582	0.216357	
x_1_:x_4_	1	21,573	21,573	17.6329	0.000162	***
x_2_:x_3_	1	2389	2389	1.9523	0.170665	
x_2_:x_4_	1	360	360	0.294	0.590914	
x_3_:x_4_	1	15,394	15,394	12.5823	0.001078	**

## Data Availability

Data available on request.
